# Potential Role of OERP as Early Marker of Mild Cognitive Impairment

**DOI:** 10.3389/fnagi.2018.00272

**Published:** 2018-09-13

**Authors:** Sara Invitto, Giulia Piraino, Vincenzo Ciccarese, Laura Carmillo, Marcella Caggiula, Giorgio Trianni, Giuseppe Nicolardi, Santo Di Nuovo, Michela Balconi

**Affiliations:** ^1^Human Anatomy and Neuroscience Laboratory, Department of Biological and Environmental Sciences and Technologies, University of Salento, Lecce, Italy; ^2^Microelectronics and Microsystems, Unite of National Research Council, Lecce, Italy; ^3^Laboratory of InterDisciplinary Research Applied to Medicine, Lecce, Italy; ^4^Istituto Santa Chiara, Lecce, Italy; ^5^Neurology Unite, Vito Fazzi Hospital, Lecce, Italy; ^6^University of Catania, Catania, Italy; ^7^Department of Psychology, Università Cattolica del Sacro Cuore, Milan, Italy

**Keywords:** CSERP, olfactory perception, MCI, neurodegenerative processes, aging, OERP

## Abstract

Olfactory impairment is present in up to 90% of patients with Alzheimer’s disease (AD) and is present in certain cases of mild cognitive impairment (MCI), a transient phase between normal aging and dementia. Subjects affected by MCI have a higher risk of developing dementia compared to the general population, and studies have found that olfactory deficits could be an indicator of whether such a conversion might happen. Following these assumptions, aim of this study was to investigate olfactory perception in MCI patients. We recruited 12 MCI subjects (mean age 70 ± 6.7 years) through the Alzheimer Assessment Unit (UVA Unite) of ASL Lecce (Italy), and 12 healthy geriatric volunteers (HS) as the control group (mean age 64 ± 6.0 years), all of whom were first evaluated via a panel of neuropsychological tests. Subjects were asked to perform an olfactory recognition task involving two scents: rose and eucalyptus, administrated in the context of an oddball task during EEG recordings. Olfactory event-related potential (OERP) components N1 and Late Positive Potential (LPC) were then analyzed as measures of the sensorial and perceptive aspects of the olfactory response, respectively. It was determined that, in the MCI group, both the N1 and LPC components were significantly different compared to those of the HS group during the execution of the oddball task. In particular, the N1 amplitude, was reduced, while the LPC amplitude was increased, indicating that a degree of perceptive compensation can occur when sensorial function is impaired. Further, a correlation analysis, involving OERP components and neuropsychological battery scores, indicated that impairment of olfactory perception may share common pathways with impairments of the spatial system and long-term memory processing.

## Introduction

Mild cognitive impairment (MCI) refers to a clinical state marking the transitional phase between normal cognitive function and pathogenic Alzheimer’s disease (AD) (Gauthier et al., 2006), characterized by deficits relating to memory, attention span, language, visuospatial ability, the speed of perception, and the performance of executive functions (Saunders and Summers, 2010; Petersen, 2011). Recent literature includes MCI due to AD (Albert et al., 2011) adding also the concept of prodromal AD (Dubois and Albert, 2004; Suk and Shen, 2013; Green et al., 2015), and differentiation between amnestic MCI (aMCI), non-amnestic MCI (naMCI) (Dubois and Albert, 2004; Csukly et al., 2016). In aMCI the memory loss is predominant and it is associated with high risk to further conversion to AD (Grundman et al., 2004). Patients with naMCI do not show memory impairment, but do display loss in other cognitive domains and have a higher risk of developing the disease in other dementia forms (e.g., Lewy Body dementia) (Csukly et al., 2016). Both categories can be further assessed to single-domain and multi-domain according to the involvement of one or more cognitive deficit (memory, language, visuospatial ability, speed of mental processing, or executive function) (Libon et al., 2010; Albert et al., 2011). The impairment of neuropsychological and psychophysiological functions can be assessed at behavioral level through neuropsychological tests and can be related with data obtained through different neuroimaging tools (MRI, PET, or SPECT) (Albert et al., 2011). One of these clinical aspects is the atrophy of the hippocampus and the entorhinal cortex, structures involved in the sense of smell (Chételat et al., 2005; Devanand et al., 2007; Shi et al., 2009; Mueller et al., 2010). Clinical studies have shown that olfactory deficits often appear very early in patients at early stages of AD and/or MCI, often before the manifestation of cognitive symptoms (Masurkar and Devanand, 2014; Roberts et al., 2016). Olfactory impairment is significantly associated with aMCI and may improve the accuracy of the model used fit to predict MCI. Furthermore it is associated with progression from MCI to dementia, and from aMCI to AD (Roberts et al., 2016). So, detection of olfactory impairment could be particularly important when investigating for cue at this neurodegenerative level, given the fact that pathological changes will have already reached the neocortex by the time AD presents itself at the clinical level (De Santi et al., 2001; Chételat et al., 2005; Roberts et al., 2016). Early detection of AD-related developments in patients with MCI provides a window of opportunity within which disease progression may be delayed or forestalled through lifestyle changes, as well as preventative measures such as enriched environment, memory and cognitive training and neurocognitive enhancement (Hinrichs et al., 2011).

During the AD progression, a progressive disruption of the cortical association areas, involved in information retrieval, and a reduction of subcortical processing, leads to a rapid loss of neurons and synapses (Tampellini, 2015). Even in the early stages of the disease, there is a sharp decline in cognitive and memory functions. Thus, prodromal AD or MCI due to AD, broadly map onto the pattern of neurofibrillary tangle spreading and is characterized by an atrophy in medial perirhinal cortex, entorhinal cortex and lateral perirhinal cortex (Braak et al., 2006; Krumm et al., 2016), cortical structures closely related to olfactory discrimination (Van Groen and Wyss, 1990; Haberly, 2001; Poellinger et al., 2001; Chapuis et al., 2013). MRI and Fludeoxyglucose-PET (FDG-PET) studies have demonstrated that olfactory performance is significantly reduced in AD patients compared to control subjects (Wesson et al., 2010; Masurkar and Devanand, 2014). In MCI difference between early and advanced stages, are seen at both functional and anatomical levels due to atrophy in several areas, such as the para-hippocampal gyrus, the medial temporal lobe, the entorhinal cortex of the cingulum, the insula, and the thalamus (Apostolova et al., 2010; Davatzikos et al., 2011), while in AD, atrophy was seen in the entire hippocampus and the neighboring regions, as well as in the temporal lobe, the cingulum, the precuneus, the insular cortex, in the caudate nucleus, and in the frontal cortex (Chételat et al., 2005; Manning et al., 2014).

Olfactory deficits have also been identified in subjects possessing ApoE e4 allele (the best-known genetic risk factor for the development of AD) (Bertram et al., 2007), independent of their current cognitive function (Manning et al., 2014) or their short-term risk of developing AD (Olofsson et al., 2010), which suggests that this gene plays an important role in olfactory identification. It should be noted that gradual anosmia can also occur in healthy elderly subjects (Bahar-Fuchs et al., 2010). Moreover, a study conducted using the University of Pennsylvania Smell Identification Test (UPSIT), a behavioral assessment of olfactory memory, concluded that olfactory deficits may be a useful biomarker of AD progression (Kirkpatrick et al., 2006), while an MRI study of the olfactory bulb and olfactory tract atrophy in MCI and AD patients has indicated that olfactory bulb atrophy could be a surrogate biomarker of AD (Graves et al., 1999). Similarly, while several groups have observed AD-specific neuropathology occurring within the olfactory epithelium (Crino et al., 1995; Masurkar and Devanand, 2014), others, using a different set of markers, have reported seeing the same in the olfactory tissues of healthy and non-AD subjects (Yamagishi et al., 1994). Furthermore, other studies have failed to identify any marker present in the olfactory epithelium that would allow AD to be reliably distinguished from other conditions, such as Parkinson’s disease or vascular dementia (Crino et al., 1995).

While several studies have described changes in event-related potentials (ERP) in MCI (Olichney et al., 2008; van Deursen et al., 2009; Chapman et al., 2011; Laskaris et al., 2013; Green et al., 2015), none so far have evaluated the use of olfactory event-related potentials (OERP) or chemosensory event-related potentials (CSERP) as tools for the investigation of the functional response to controlled chemical stimulation in MCI. We contend that the peculiarities of these ERPs could be exploited to gain a better understanding of the brain areas associated with the processing of various stimuli (e.g., visual, auditory, nociceptive, etc.), and the temporal latencies that are involved. In the present study, we investigate the OERP latencies and amplitudes relating to the sequential activation of different brain areas, starting with the olfactory bulbs, and progressing through the frontal and insular orbital cortex and the middle-rostral regions of the temporal lobe.

Olfactory event-related potentials consist of an early negative N1 component, followed by a positive phase termed P1, or the Late Positive Component (LPC) (Hummel et al., 1998; Kobal and Hummel, 1998; Pause and Krauel, 2000; Gudziol et al., 2014). When an odoriferous molecule activates the olfactory cells, a negative potential is generated, which is followed by a rebound potential which can be measured by placing electrodes near the olfactory epithelium (Lötsch and Hummel, 2006); the dimension of the track that reproduces the potentials generated change with the variation of the stimulus concentration and shows some evidence of the adaptation phenomenon (Pause and Krauel, 2000; Wang et al., 2002).

Usually, during OERP recordings subject had to perform a simple tracking task or a simple olfactory recognition task (Pause et al., 1996; Lötsch and Hummel, 2006; Invitto et al., 2018). The nasal stimulation can be left or right lateralized (Pause et al., 1996) or frontal (with the stimulation of both the nostrils) (Invitto et al., 2018). As usually is for other ERP component early negative component (i.e., N1) is an indicator of the cortical sensorial response, and late components (i.e., LPC) are indicators of the cortical perceptive and cognitive responses to the stimuli. The present research aims to assess if there are changes in OERP that can be correlated with perceptual impairments in MCI patients, and if these changes can be helpful to investigate impaired multi-domain cognitive components.

## Materials and Methods

This research was conducted in the Neurology Unit of the Vito Fazzi Hospital (Lecce, Italy). Data collection was performed in accordance with the Code of Ethics of the World Medical Association (Declaration of Helsinki), and written informed consent was obtained from all participants. The study protocol was approved by the Ethical Committee of Vito Fazzi Hospital, Lecce (Report No.01 – 30-01-17).

### Subjects

This study involved 12 geriatric MCI patients and 12 healthy geriatric subjects (HS) recruited between February 2017 and January 2018, matched by age, gender and education. Patients (mean age 70.25 years; SD ± 7.74) were enrolled with a clinical suspicion of MCI and admitted to the Alzheimer Assessment Unit (UVA Unite; Lecce, Italy) of the ASL Lecce (Italy), where neuropsychological and olfactory psychophysiological evaluations were conducted. MCI diagnosis was confirmed by neurological and neuropsychological assessment, according to NINCDS-ADRDA (McKhann et al., 1984; Dubois et al., 2007), DSM-V guidelines (American Psychiatric Association, 2013) and according to the latest guidelines and recommendation of National Institute on Aging Alzheimer’s Association (NIA-AA) (Albert et al., 2011). According to NIA-AA guidelines our MCI sample can be linked to an intermediated likelihood that MCI syndrome is due to AD. Infact, all patients showed positive biomarkers for neuronal injury (i.e., hippocampal or medial temporal lobe atrophy, diffuse cortical atrophy on MRI and so on). Furthermore, the standard hematochemical tests carried out on these patients, excluded, together with the MRI, the presence of other pathologies and or co-morbidities (Musicco et al., 2004). During the recruitment phase, the patients with other forms or causes of dementia (Babiloni et al., 2016), determined by anamnestic analysis, were excluded from the study.

HS (mean age 66.41 years; SD ± 5.71) were enrolled as volunteers through recruitment notice provided through university students. The HS did not report any current or past psychopathology, neurological illness, or substance abuse and did not report any impairment in normal daily activities.

### Neuropsychological Assessment

Subjects (HS and MCI) were scored through the Mini-Mental State Examination (MMSE), Trial Making Test (TMT), Corsi Test (CT), Digit Span (DS), and Rey Auditory Verbal Learning Test (AVLT) according to NIA-AA guidelines (Albert et al., 2011) and to the guideline of Italian Society of Neurology (Musicco et al., 2004) (see neuropsychological scores in **Table 1**). Via *t*-tests, it was determined that while the two groups were not appreciably dissimilar in their ages and gender compositions (*p* > 0.05), their MMSE scores were significantly different, as was to be expected (*p* < 0.05).

**Table 1:** MMSE, Rey Auditory Verbal Learning Test (AVLT), Digit Span Test, Corsi Spatial Test, and Trial Making Test (TMT) scores.

SUBJ	AGE range	MMSE	AVLTa	AVLTb	AVLTc	Digit Span	Corsi Test	TMT	TMT-B	TMT-AB	ADL	IADL
MCI	75–80	<22	0	0	0	3	1	0	0	0	6	8
MCI	55–60	<22	3	3	15	4	4	1	1	1	6	8
MCI	75–80	<22	3	4	15	3	3	2	1	4	6	8
MCI	70–75	<22	0	0	7	4	4	0	1	2	6	8
MCI	65–70	<22	0	0	7	3	3	0	1	4	6	8
MCI	75–80	<22	1	1	6	4	3	0	0	1	6	8
MCI	60–65	<22	1	0	0	4	2	4	3	3	6	8
MCI	70–75	<22	1	0	0	4	1	0	1	4	6	8
MCI	60–65	<22	0	2	7	3	4	0	1	4	6	8
MCI	65–70	<22	0	0	7	2	2	2	1	1	6	8
MCI	80–85	<22	3	3	15	1	4	1	1	1	6	7
MCI	70–75	<22	1	0	15	1	1	0	0	0	6	8
HS	60–65	>24	25	5	15	6	5	4	4	4	6	8
HS	65–70	>24	27	13	15	5	6	4	4	4	6	8
HS	70–75	>24	46	11	15	6	4	4	4	4	6	8
HS	65–70	>24	50	15	15	7	5	4	4	4	6	8
HS	75–80	>24	48	14	15	6	6	4	4	4	6	8
HS	65–70	>24	32	15	15	5	5	4	4	4	6	8
HS	65–70	>24	30	12	15	6	7	4	4	4	6	8
HS	70–75	>24	41	11	15	5	6	4	4	4	6	8
HS	60–65	>24	25	15	15	6	7	4	4	4	6	8
HS	60–65	>24	21	14	15	5	7	4	4	4	6	8
HS	55–60	>24	41	11	15	6	6	4	4	4	6	8
HS	70–75	>24	22	12	15	5	5	4	4	4	6	8


### Olfactory Psychophysiological Assessment

Subjects performed an olfactory recognition task involving two scents: rose odor perception (β-PEA, 2-Phenylethanol, CAS Number: 60-12-8, Number W285803 Sigma-Aldrich) and eucalyptus odor perception (1,3,3-Trimethyl-2-oxabicyclo [2.2.2] octane, CAS Number: 470-82-6, Number C80601 Sigma-Aldrich) chosen according to Hummel et al. (2009). Scents were administered via US2017127971 (A1) 2017-05-11 (Invitto et al., 2014), with 20 μL PEA provided in 10 mL of Vaseline oil, and both odorous solutions were presented in 20 mL transparent glass vials. Both scents were sealed with plastic film and stored in a darkened cabinet.

The presentation paradigm was an oddball task (Squires et al., 1975) adapted to olfactory stimulation (Invitto et al., 2018). Oddball olfactory task consisted in a pseudorandom administration of different smells (i.e., two different odorants) one of which is the rare stimulus (target stimulus) and the other is the frequent (non-target) stimulus. Usually the percentage of presentation of the rare stimulus is 25%. The presentation of the stimuli is pseudo-randomized so that the subject cannot predict the sequence of stimuli administration. Each stimulation had a duration of 450 ms, with an interstimulus interval (ISI) of 60 s, to avoid olfactory habituation (Pause and Krauel, 2000). The task ended after 40 min.

The device used to record odorous stimuli presentation allows the CSERPs evoked by olfactory stimuli to be measured in a controlled, automated fashion, synchronized to the acquisition of the EEG signal. This method additionally allowed for the blind presentation of smells (Invitto et al., 2014, 2017b).

### OERP Recording

**FIGURE 1:**
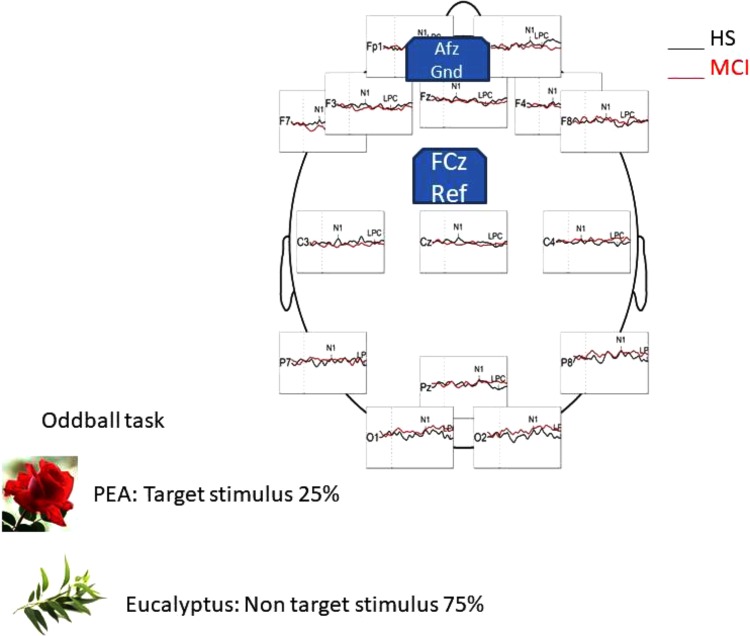
Head representation illustrating the primary EEG montage used for recording during the oddball task in HS group, with a general comparison of OERP component generated by the PEA (rose odorant) stimulus.

EEG signals were recorded using a 16-channel amplifier (Brain Products V-Amp), mounted on an electrode cap equipped with Ag/AgCl electrodes. Brain Vision Recorder and Brain Vision Analyzer (Brain Products GmbH) analysis software were used for the OERP study. Electrode impedance was kept below 15 kΩ, and the EEG recording sampling rate was 500 Hz. Electrodes were online referenced to FCz (Luck, 2005), and offline re-referenced with a common offline reference over all electrodes (Luck, 2005). One electrode was placed at the outer canthus of the right eye and used to monitor eye movements. Trials contaminated by eye movements and other artifacts were rejected. The signal was filtered offline (0.01–50 Hz, 24 dB), and the threshold for artifact rejection was set at >|125| μV (Pause et al., 1996, 1997). Ocular rejection was performed through independent component analysis (ICA). ERP epochs included a 100 milliseconds pre-stimulus baseline period and a 500 milliseconds post-stimulus segment. Separate averages were calculated for each odorant segmentation (rose and eucalyptus). The detection of peaks was performed as previously described (Invitto et al., 2017a). OERP components were labeled N1 and LPC according to Pause et al. (1996). Latency windows were set to 150–300 ms for the N1, and 300–500 ms for the LPC (Pause et al., 1996; Pause and Krauel, 2000). These values correspond to the onset of CSERP negative and positive peaks components estimated from grand average waveforms (see **Figure 1**).

Moreover, we performed a Current Source Density (CSD), a topographic representation of EEG voltage values across the scalp. CSD is the results of mathematical algorithms, in this research conducted through the Brain Vision Analyzer software, that directly transform the scalp-recorded EEG into estimates of radial current flow at scalp. So, in EEG-CSD topography, the positive values identify the direction between the brain source to the scalp, and negative values represent current flow from the scalp to the brain source (Kayser et al., 2010; Kamarajan et al., 2016). To obtain CSD images, grand average OERP waveforms at each electrode were transformed into reference-free CSD estimates using a spherical spline surface Laplacian (interpolation of Spherical Splines – order of Splines 4; Max degree of Legendre Polynomial: 10; Default Lambda 1e-5) (Perrin et al., 1989; Kayser et al., 2010). The number of two representation Maps were chosen in the Interpolation window and the interval between Maps (ms/Hz) was defined as indicated in the default value (1000). The selected range of maps visualization was set between 100 and 500 ms (i.e., the temporal range in which the two OERP components were detected).

Then a method comparison operation was carried out in a subtractive way (i.e., differences between different datasets), by subtracting between frequent and infrequent stimulus, to topographically view the generic attentional effect rather than the olfactory oddball task due to the administration of two different odors.

### Statistical Analysis

All statistical analyses were performed using IBM SPSS version 20. Further details regarding statistical tests are described in the following section.

## Results

**FIGURE 2:**
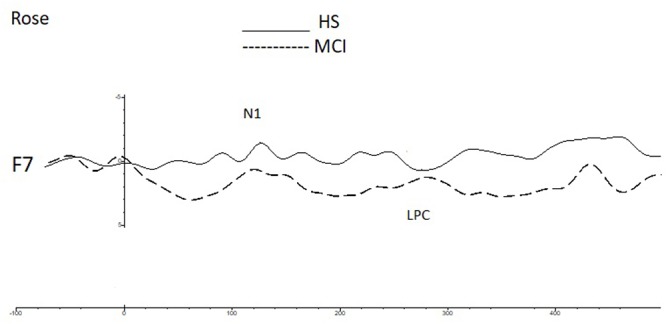
A comparison of the grand averages of OERP waveforms (recorded at the F7 electrode) elicited by the rose odorant for the MCI (dashed line) and HS (continuous line) groups. The N1 and LPC components are labeled.

Shapiro-Wilk and Kolmogorov-Smirnov tests showed that the HS and MCI groups behaved differently from each other with regards to the amplitudes of both the N1 and LPC components. Consequently a separate non-parametric one way ANOVA (i.e., Kruskal-Wallis Test for independent samples) was used for group comparisons (Maris and Oostenveld, 2007). We first analyzed the amplitudes of the N1 components elicited by the rose odorant using the Kruskal-Wallis test. Significant differences were observed at the F3 position (*z* = 4.452; *p* = 0.03), with the MCI group showing decreased N1 amplitudes [MCI mean = -2.85 μV (SD = 3.39) vs. HS mean = -5.02 μV (SD = 3.13)], at F7 (*z* = 9.200; *p* = 0.002), where N1 was also smaller in the MCI group [MCI mean = -2.63 μV (SD = 1.60) vs. HS mean = -6.49 μV (SD = 3.94)] (see **Figure 2**) and at F8 (*z* = 5.635; *p* = 0.018), where N1 amplitude was higher in the MCI group [MCI Mean = -5.72 μV (SD = 3.96) vs. HS mean = -1.96 μV (SD = 3.23)]. There was, however, no significant difference in the N1 latencies of the two groups. Analysis of LPC amplitudes for the rose odorant identified a significant difference at the F7 (*z* = 4.278; *p* = 0.04) and F8 (*z* = -4.839; *p* = 0.028) positions, with the MCI group exhibiting enhanced amplitudes at the former [MCI mean = 8.74 μV (SD = 3.89) vs. HS mean = 3.79 μV (SD = 3.89)], and decreased amplitudes at the latter [MCI mean = 5.51 μV (SD = 2.50) vs. HS mean = 9.72 μV (SD = 4.52)] (a comparison of OERP components is shown in **Figure 1**). The mean amplitudes (±sSD) of OERPs elicited by the rose odorant for HS and MCI groups are shown in **Table 2**.

**Table 2:** Means of N1 and LPC amplitudes (μV) elicited by the rose odorant for MCI and HS groups.

Electrode	N1 Rose					LPC Rose				
		
	HS		MCI			HS		MCI		
				
	Mean	*SD*	Mean	*SD*	*p*	Mean	*SD*	Mean	*SD*	*p*
Fp1	-5.58	6.63	-3.55	4.12	0.42	3.91	6.29	5.54	2.45	0.53
Fp2	-5.12	7.24	-4.11	2.83	0.60	5.31	6.66	4.75	2.67	0.77
F3	-5.02	3.13	-2.85	3.39	0.03^∗^	4.13	5.53	5.54	3.98	0.22
F4	-3.53	1.52	-4.42	2.10	0.21	5.39	2.61	4.51	2.69	0.45
C3	-4.29	3.58	-2.47	1.88	0.29	2.69	3.43	4.05	3.49	0.77
C4	-3.34	2.35	-4.56	3.41	0.74	3.55	3.03	3.00	2.89	0.58
P7	-6.22	5.59	-5.04	3.01	0.64	6.23	3.69	6.25	2.60	0.77
P8	-6.45	3.73	-4.95	3.84	0.26	6.74	5.09	5.70	3.30	0.20
O1	-6.24	4.92	-6.42	2.99	1.00	5.67	3.69	5.12	3.38	0.87
O2	-5.88	4.53	-5.96	3.03	0.89	6.86	2.88	5.37	3.35	0.25
F7	-6.49	3.94	-2.63	1.60	0.00^∗^	3.97	3.89	8.74	6.79	0.04^∗^
F8	-1.96	3.23	-5.72	3.96	0.01^∗^	9.72	4.52	5.51	2.50	0.03^∗^
Cz	-3.76	2.91	-2.37	0.79	0.13	2.49	3.36	3.13	2.68	0.77
Pz	-3.74	3.44	-3.60	1.98	0.64	4.01	3.00	4.61	2.91	0.92
Fz	-2.48	1.99	-2.36	2.91	0.94	4.77	3.87	3.97	2.64	0.72


*SD, standard deviation; p, p-value.*

**FIGURE 3:**
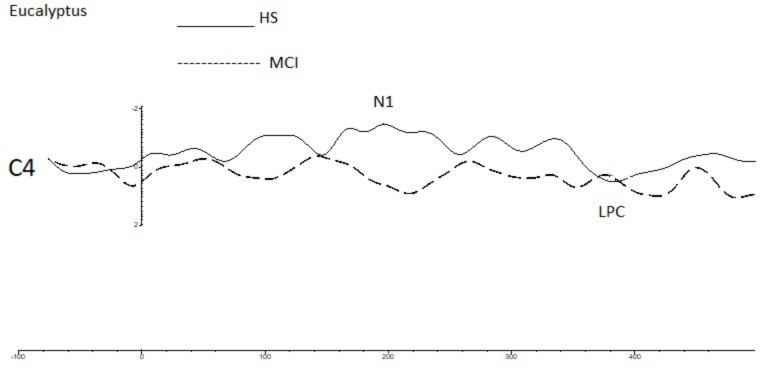
A comparison of the grand averages of OERP waveforms (recorded at the C4 electrode) elicited by the rose eucalyptus for the MCI (dashed line) and HS (continuous line) groups. The N1 and LPC components are labeled.

To investigate overall trends in olfactory perception of frequently encountered stimuli in an oddball setting, we analyzed the OERPs elicited by eucalyptus. The Kruskal-Wallis test for independent samples revealed significant differences in N1 amplitudes at C4 (*z* = 7.934; *p* = 0.005), where values were lower in the MCI group [MCI mean = -1.45 μV (SD = 0.76) compared to HS mean = -3.30 μV (SD = 1.75)], F7 (*z* = -5.078; *p* = 0.024) [MCI mean = -4.29 μV (SD = 2.40) vs. HS mean = -1.91 μV (SD = 2.15)], and in N1 latencies at Fp2 (*z* = 5.084; *p* = 0.024) [MCI mean = 157 ms (SD = 60) vs. HS = 215 ms (SD = 45)] and F7 (*z* = 5.081; *p* = 0.024) [MCI mean = 211 ms (SD = 48) vs. HS mean = 162 ms (SD = 63)]. Detailed features of the N1 and the LPC are shown in **Table 3**, and a comparison of HS and MCI OERPs recorded at the C4 electrode is shown in **Figure 3**. Our analysis also indicated that there were no significant differences in the LPC components elicited by the eucalyptus scent.

**Table 3:** Means of N1 and LPC amplitudes (μV) elicited by the eucalyptus odorant for MCI and HS groups.

Electrode	N1 eucalyptus					LPC eucalyptus				
		
	HS		MCI			HS		MCI		
				
	Mean	*SD*	Mean	*SD*	*p*	Mean	*SD*	Mean	*SD*	*p*
Fp1	-3.58	4.36	-2.82	2.41	0.89	3.21	4.47	3.45	2.84	0.57
Fp2	-4.49	4.85	-2.44	3.01	0.14	3.15	3.38	3.72	2.12	0.62
F3	-2.43	1.71	-3.56	1.46	0.14	3.45	2.26	2.53	1.15	0.57
F4	-2.98	1.07	-1.87	1.52	0.91	2.76	1.86	4.52	2.53	0.12
C3	-2.13	0.91	-2.61	1.11	0.26	2.17	1.16	2.22	0.96	0.72
C4	-3.30	1.75	-1.45	0.76	0.00^∗^	1.97	1.45	2.76	1.87	0.29
P7	-2.90	2.76	-3.83	1.90	0.44	5.24	2.95	3.37	2.23	0.18
P8	-3.64	3.46	-2.99	1.86	0.78	2.89	3.16	4.47	3.25	0.26
O1	-3.11	3.27	-5.60	2.98	0.08	5.50	4.49	4.30	5.41	0.48
O2	-3.45	3.52	-4.61	3.07	0.53	4.46	3.83	5.18	3.86	0.67
F7	-1.91	2.15	-4.29	2.41	0.02^∗^	4.35	3.19	3.17	2.83	0.52
F8	-3.17	2.07	-2.72	3.18	0.48	4.01	2.41	5.15	2.16	0.12
Cz	-2.37	0.91	-1.66	1.31	0.16	2.37	1.15	2.83	1.81	0.52
Pz	-2.03	1.96	-2.83	1.48	0.08	3.55	2.22	3.18	2.54	0.29
Fz	-2.82	2.78	-2.31	1.88	0.89	2.67	2.11	2.89	1.82	0.94


*SD, standard deviation; p, p-value.*

**FIGURE 4:**
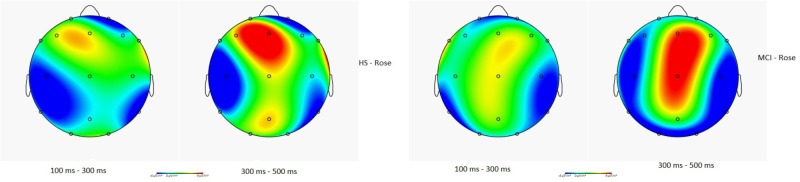
Topographical map of current source density in the N1 and LPC intervals in the HS and MCI group when presented with the rose odorant.

**Figure 4** illustrates the CSD in HS and in MCI groups for the rose odorant, during the N1 and LPC phases. Images show how the two samples allocate the attention differently. In general, the range in voltage is lower in the MCI (MCI = ± 2.03 μV vs. HS = ± 2.45 μV) and the negative component seems to be more frontal and lateralized on the left, while the positive component seems more lateralized on the right occipito-temporal region.

The association between the results of the neuropsychological test and the OERP amplitudes that had been found to be significantly different between MCI and HS groups via the analyses described above (i.e., those recorded at F3, F7, and F8 for the N1 component and F7 and F8 for the LPC component) was further investigated via Kendall’s tau-b (τb) correlation coefficient analysis, for which detailed values are shown in **Table 4**. A negative correlation between the N1 amplitudes of F7 and F3 was seen. Additionally, F7 N1 amplitude was found to be positively correlated with high neuropsychological test scores, and an association between the CORSI and TMTs was also seen (A-B-AB). Moreover, OERP amplitudes were found to be related to spatial scores. While left frontotemporal components were determined to be negatively correlated with high scores in spatial memory tasks, right frontotemporal components were, by contrast, positively correlated with the same. Finally, we also observed that subject scores for the Rey AVLT, which tests for episodic declarative memory, are particularly highly correlated with scores achieved in the b version of the test, designed to evaluate long-term episodic memory.

**Table 4:** Kendall’s tau-b correlation coefficients relating neuropsychological test scores and OERP amplitudes significantly affected in the MCI group (F3, F7, and F8 in N1 component and F7 and F8 in LPC component).

	KENDALL’S TAU-B									
	
	F3 (N1)		F7 (N1)		F8 (N1)		F7 (LPC)		F8 (LPC)	
					
	Cor.	*p*	Cor.	*p*	Cor.	*p*	Cor.	*p*	Cor.	*p*
MMSE	-0.23	0.15	-0.35	0.03ˆ*	0.24	0.14	-0.22	0.17	0.31	0.05ˆ*
AVLTa	-0.21	0.20	-0.33	0.05ˆ*	0.31	0.06	-0.17	0.29	0.25	0.13
AVLTb	-0.34	0.05ˆ*	-0.54	0.00ˆ**	0.44	0.01ˆ*	-0.40	0.02ˆ*	0.38	0.03ˆ*
AVLTc	-0.32	0.05ˆ*	-0.33	0.04ˆ*	0.19	0.26	-0.26	0.11	0.35	0.03ˆ*
Digit Span	-0.14	0.39	-0.38	0.02ˆ*	0.42	0.01ˆ*	-0.32	0.06	0.26	0.12
Corsi	-0.25	0.12	-0.46	0.00ˆ**	0.31	0.06	-0.38	0.02ˆ*	0.37	0.02ˆ*
TMT	-0.33	0.06	-0.52	0.00ˆ**	0.31	0.09	-0.29	0.10	0.40	0.02
TMT-B	-0.33	0.05ˆ*	-0.52	0.00ˆ**	0.36	0.04ˆ*	-0.41	0.02	0.43	0.01ˆ*
TMT-AB	-0.26	0.13	-0.40	0.02ˆ*	0.36	0.04ˆ*	-0.45	0.01ˆ**	0.29	0.10


## Discussion and Conclusion

Olfaction can serve as a useful biomarker in processes such as AD and Parkinson’s disease, as it is often the first sense to be affected by neurodegeneration. It is additionally known that subjects afflicted with MCI and/or who possess the e4 allele of the ApoE gene, which has been linked to an increased risk for the development of AD, have severe anosmia (Olofsson et al., 2010). Further, MRI studies have suggested that the olfactory bulb is impaired in patients with MCI and AD (Wesson et al., 2010). Finally, several groups have described changes in ERP in MCI (Olichney et al., 2008; van Deursen et al., 2009; Chapman et al., 2011; Laskaris et al., 2013; Green et al., 2015). However, studies relating to OERPs in MCI or AD are currently lacking.

**FIGURE 5:**
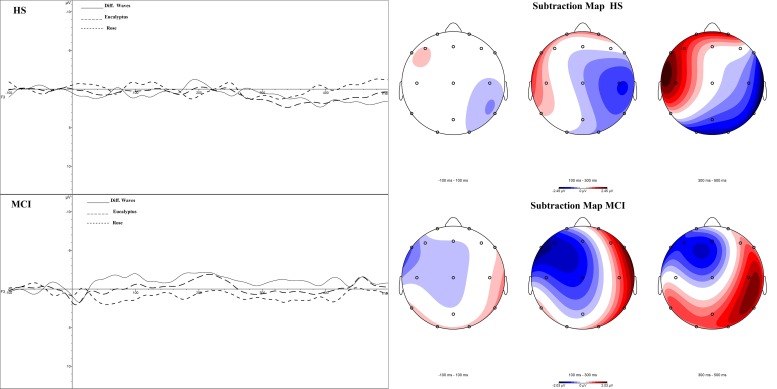
Subtraction OERP components and Map in HS and MCI patients.

Compared to techniques such as MRI or fMRI, OERP measurements also have a higher temporal resolution, and can be conducted at lower cost with a lower degree of invasiveness. We have, for these reasons, developed a research protocol designed to assess both the sensorial and perceptual aspects of the olfactory cortical response in MCI patients (i.e., sample with intermediate probability that MCI is due to AD). In this study, we evaluated various facets of both the early (N1) and slow potentials (LPC) of the OERP in MCI patients and HS to determine if significant differences exist between the two groups (**Figure 5**). Our results showed a clear deficit in the early sensory N1 component in the MCI group, which exhibited reduced amplitudes in the left frontal and right centroparietal lobes compared with healthy controls, which may indicate reduced olfactory discrimination. Topographic mapping of OERP intensities across the scalp showed that the negative components are more compromised in the left orbitofrontal and frontotemporal areas and in the right centroparietal area, which are particularly closely associated with olfactory OERPs (Schriever et al., 2014). Also, slow positive potentials in the left prefrontal cortex in the MCI group had greater amplitudes, and longer latencies. This brain area is involved in olfactory perception and memory, and is specifically activated during the administration of pleasant scents (Soudry et al., 2011). The increased amplitude could be a mechanism compensating for impairments in the olfactory-sensorial components (i.e., N1), indicating the existence of a feedback system capable of activating more arousing resources to better detect the olfactory stimulus (e.g., LPC could be a component that can covert cognitive resources).

A similar mechanism was observed in a previous study involving obstructive sleep apnea syndrome (Invitto et al., 2018). However, the greater activation of early OERP components in the right frontoparietal area and the reduction of LPC in MCI patients seen here can be extremely important in supporting the previous results. Studies have demonstrated that the right frontotemporal area is responsible for the recruitment of olfactory memory rather than its sensorial perception. Also this activation can be the consequence of a compensation of a sensory deficit which, to be efficient, relies on more mnestic than perceptive components (Jones-Gotman and Zatorre, 1993). Further specifications can be due to the correlation between neuropsychological test and OERP results. The best area that fit with olfactory responses and neuropsychological scores is the left frontotemporal area (high scores to neuropsychological tests are related with greater negative amplitude in N1). This area is strongly associated with odor discrimination (Rami et al., 2007). Another interesting result from this study is the correlation between CORSI and TMT scores. OERP amplitudes are related to spatial scores. Left frontotemporal components have negative correlations with high scores in spatial memory tasks, whereas, right frontotemporal components have a positive correlation. These observations support the existing paradigm that the spatial and olfactory systems are closely associated at the level of the cortical pathways (Jacobs et al., 2015). The Rey AVLT, a test for episodic declarative memory, is highly correlated, in particular, the scores of the section linked to long-term episodic memory.

Our data demonstrate the usefulness of OERPs in the study of the early stages of neurodegenerative processes, as there was a clear defect in olfactory function in MCI patients, confirming the findings of others. One particular area that still needs further investigation is to clarify the extent that MCI samples are caused by AD. In fact, our sample was not recruited after genetic or CSF diagnosis, which would confirm AD pathology underlying the disease or the prodromal AD (Dubois and Albert, 2004). On the other hand, this study also did reveal the existence of compensatory processes that are activated to balance these deficits during the early stages of multi-domain aMCI, involving not only the sensory system, but also the spatial system. This process seems linked to the recognition process through long-term memory, which, in the specifics of olfactory perception, seems to be activated to recognize the odors no longer through its sensorial components, but through long-term recognition features. We intend to further develop this research by reassessing the same functions within the same cohort of subjects, over the next year, in order to determine if MCI has developed into AD in any of the cases. Our follow up will be conducted after adequate tests assessment, including neuroimaging, biomarkers and genetic assessment of ApoE gene. So we will examine how the olfactory components have been affected by the neurodegenerative process. This will allow us to understand whether these electrophysiological findings found in the multimodal amnesic MCI (with intermediate probability that MCI is due to AD) remain constant or progress; we will also understand if the subjects enter a diagnostic phase of MCI due to AD or we may actually find direct evidence in AD.

In any case, the electrophysiological data produced by olfactory stimulation shows us that in MCI there are complex alterations that also provide compensatory mechanisms with respect to the physiological responses of geriatric control subjects. These compensatory mechanisms can be considered as further cues in a diagnostic border picture, such as that of MCI with intermediate connection to AD, which shows altered responses on various levels. The assessment of a low-cost, relatively fast and non-invasive test such as that of OERPs may be an additional data to be integrated into this particular diagnostic frame.

## Author Contributions

SI contributed to research conception, protocol design, subjects EEG recording, EEG and neuropsychological data analysis, paper writing, and paper review. GP Neuropsychological Tests Administration and scoring. MC, LC, and GN MCI assessment and recruitment. VC technical support to olfactometer. GT Head of Neurology Unite – supervision on patients assessment and recruitment. SDN neuropsychological scoring analysis. MB theoretical suggestions in the paper review.

## Conflict of Interest Statement

The authors declare that the research was conducted in the absence of any commercial or financial relationships that could be construed as a potential conflict of interest.
